# Chk1 activity is required for BAK multimerization in association with PUMA during mitochondrial apoptosis

**DOI:** 10.1186/s12964-014-0042-1

**Published:** 2014-07-10

**Authors:** Abul Azad, Alan Storey

**Affiliations:** 1 Department of Oncology, The Weatherall Institute of Molecular Medicine, University of Oxford, Oxford OX3 9DS, UK

**Keywords:** Mitochondria, Apoptosis, BAK, BCL-2, BH3, Chk1, DNA damage

## Abstract

**Background:**

The Bcl-2 protein BAK is a key player in mitochondrial apoptosis and responds to a myriad of different death signals. Activation of BAK is a multistep process that involves a number of conformational changes mediated by BH3-only proteins or p53 which leads to BAK multimerization and pore formation in the mitochondrial outer membrane. We previously reported that BAK activation is dependent upon dephosphorylation of both tyrosine and serine residues. Further, recent reports demonstrated that PP2A activity is required for BAK multimerization. Since Chk1, a checkpoint kinase involved in the activation of G2 checkpoint, is regulated by PP2A, we therefore hypothesized that Chk1 is involved in BAK multimerization during cell cycle arrest upon severe DNA damage.

**Findings:**

We now show that treatment of HCT116-WT BAK cells with a Chk1 inhibitor impaired BAK dimerization and mutimerization when treated with the DNA damaging agents UV or etoposide. As a result there is a concomitant decrease of cytochrome c release from isolated mitochondria challenged with tBid protein and failure in the activation of caspase3. Interestingly, co-immunoprecipitation studies suggest that Chk1 is required for recruitment of BH3- only protein PUMA to BAK. We also showed that Chk1 is associated with BAK upon DNA damage.

**Conclusion:**

These findings novelly demonstrate the involvement of a checkpoint kinase Chk1 is required for BAK activation and underscores the importance of involvement of Chk1 in mitochondrial apoptosis upon severe DNA damage.

## Findings

Apoptosis block is a hallmark of cancer and may contribute to aggressive tumour progression. The Bcl-2 family proteins regulate the mitochondrial pathway of apoptosis through interactions between the pro-apoptotic proteins BAK and BAX, the anti-apoptotic Bcl-xL and MCL1, and the BH3-only proteins such as Bid, Bim, PUMA which activate BAK and BAX and or inhibit antiapoptotic proteins [1]. BAK activation is a complex multistep process. Upon apoptotic stimuli BAK undergoes a N-terminal conformational change initiated by a ‘hit and run’ either by BH3-only proteins or p53. This is followed by transient exposure of BAK BH3 domain which then binds to the hydrophobic groove of another BAK molecule leading to dimer formation [2],[3]. The resulting symmetric homodimers then multimerize to higher order structures via α6:α6 helices [4] leading to the permeabilization of the mitochondrial outer membrane followed by the release of apoptogenic factors like cytochrome c, that in turn lead to downstream activation of caspase3. Recently a unified model has been proposed that suggests different affinities of BH3 proteins for both pro- and anti-apoptotic proteins [5].

Together with the well-characterised conformational changes required for BAK activation we recently reported that specific dephosphorylation events are also required. Firstly, tyrosine dephosphorylation at 108 (Y108) in BAK is required to convert BAK into an ‘activation-competent’ form which constitutes an obligatory first step in BAK activation [6]. Secondly, we found that dephosphorylation at S117 residue by PP2A was required both for the BAK BH3 domain to gain access to BAK’s hydrophobic groove and permit BAK dimerization and subsequent multimerization [7]. More recently we have shown that dephosphorylation at tyrosine 110 (Y110) is required for recruitment of Bid to BAK [8].

In response to DNA damage, normal cells arrest in G1 to allow time for DNA to repair or they proceed to apoptosis if the DNA damage is too severe. Robust genotoxic insults induce apoptosis rather than the cell cycle checkpoint. Checkpoint kinase Chk1, a serine/threonine kinase that was first identified in fission yeast as an essential component of the DNA damage checkpoint [9],[10] and a major effector of G2/M-phase DNA damage checkpoint and S-phase replication checkpoint [11]. Recently it has been reported that anti-apoptotic BCL-2 family protein MCL-1 regulates Chk1 [12]. However the role of Chk1 in mitochondrial apoptosis remains to be elucidated. PP2A is known to maintain a regulatory circuit with checkpoint kinase Chk1 by continuously dephosphorylating Chk1 which is itself continuously phosphorylated by ATR (Ataxia-telangiectasia-related) [13]. Since we found that PP2A activity was required for BAK multimerization, one particular focus was to elucidate whether Chk1 activity is involved in BAK multimerization during mitochondrial apoptosis in response to DNA damage. In the present study we found that Chk1 played a role in mitochondrial apoptosis.

### Chk1 is involved in BAK multimerization upon UV irradiation

**Figure 1:**
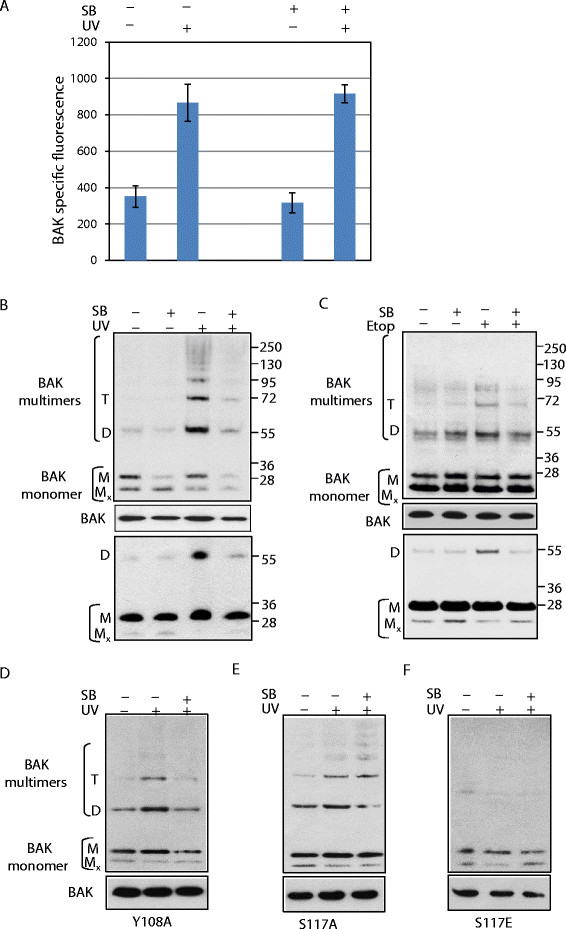
**Effect of Chk1 inhibitor on BAK activation following DNA damage. (A)** BAK N-terminal conformational change was analysed in HCT116-WT BAK cells by FACS using AB1 epitope that specifically recognizes the N-terminal open conformation of BAK. BAK underwent N-terminal conformational change causes increase in BAK AB-1 specific fluorescence after UV damage that was not inhibited by prior treatment with Chk1 inhibitor SB-218078 (Calbiochem, n = 3, ± s.e.m.). **(B & C)** BAK multimerization assay was analysed in HCT116 DKO cells reconstituted with WT BAK as described in [6]. Mitochondria were isolated from cells treated with or without Chk1 inhibitor (SB −218078) for 30 min either alone or UV **(B)** radiation or etoposide treatment **(C)** for 8 hrs. 70–100 μg of mitochondria were crosslinked with 10 mM BMH (top panel) or 10 mM BMOE (bottom panel). BAK was detected by rabbit anti BAK monoclonal ab (abcam Y164). The input was the 5% of mitochondrial extracts used in the crosslinking studies to ensure equal loading (middle panel). Non cross-linked BAK runs as a monomer (M) and also as an intra-molecularly linked monomer (Mx). BAK dimers (D), trimers (T) and higher order structures are indicated. Note that BMOE only detects monomer and dimer forms of BAK. This is representative of 3 independent experiments. Similarly, multimerization experimemts were performed with BAK Y108A mutant **(D)**, BAK S117A mutant **(E)** and BAK S117E **(F)** mutant in the presence of the Chk1 inhibitor and ± UV.

In our recent reports we showed that PP2A activity was required for mediating BAK activation [7]. Since PP2A has a regulatory role on Chk1, we reasoned that Chk1 may be involved in BAK activation during cell cycle arrest upon severe DNA damage. We investigated the role of Chk1 in BAK activation during mitochondrial apoptosis using the selective Chk1 inhibitor SB-218078, which potently inhibits Chk1 activity [14], in HCT116 cells lacking both BAK and BAX expression and reconstituted with WT BAK (HCT116-BAK cells). BAK activation consists of two major steps: an N-terminal conformational change followed by dimerization and subsequent multimerization. We first analysed the N-terminal conformation change by a FACS-based assay to determine whether SB-218078 could block BAK N-terminal conformation change when cells were treated with the inhibitor followed by UV damage. We found that the Chk1 inhibitor had no effect on BAK N-terminal conformational change (Figure 1A). We next analysed whether the Chk1 inhibitor had any impact on BAK multimerization. Mitochondria were prepared from cells treated with inhibitor and subjected to the sulfahydryl cross-linker BMH (1,6-bismaleimidohexane, Pierce). Following UV damage or etoposide treatment alone BAK readily multimerizes to higher order structures. Prior treatment with the inhibitor results in a significant reduction of BAK multimerization (Figure 1B and C). As we noticed before [7],[8] the BMH crosslinker reduced the amount of monomeric BAK even though the total input levels are the same. To overcome this problem we used a second sulfhydryl crosslinker, BMOE (Bismaleimidoethane) that is known to produce only the dimmer form of BAK rather than multimers [2],[7],[8]. Using the same mitochondrial preparations, SB-218078 was able to reduce BAK dimerization in response to either UV or etoposide treatment. Previously, we reported two different phosphorylation mutants of BAK Y108A, involved in the initial step of BAK activation, that is, an N-terminal conformational change [6], and BAK S117A/S117E, which was required for BAK multimerization [7]. Therefore, to further understand the importance of Chk1 activity in BAK multimerization, we next investigated the effect of the Chk1 inhibitor on BAK multimerization with these two phosphorylation mutants of BAK Y108A and S117A/S117E. As expected, we found that prior treatment with the Chk1 inhibitor results in inhibition of BAK multimerization with the Y108A mutant further in line with the findings that Chk1 activity is not required for the initial step of BAK activation. The Chk1 inhibitor has no impact on BAK multimerization of either S117A, which is refractory to the Chk1 inhibitor, or the S117E mutant in which BAK multimerization was severely impaired. Thus, this finding demonstrates that Chk1 activity is required for BAK activation at a later stage.

### Chk1 activity is required for cytochrome c release and caspase3 activation after UV damage

**Figure 2:**
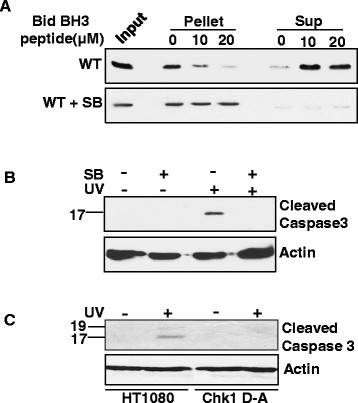
**Effect of Chk1 inhibitor on cytochrome c release from mitochondria and caspase activation. (A)** Mitochondria were isolated from HCT116-WT BAK cells and HCT116-WT BAK cells treated with Chk1 inhibitor (SB-218078) for 30 min. Mitochondria were then incubated with increasing concentrations (0.5-1 ng/μl) of recombinant tBid (R&D) at 37°C for 30 min. Mitochondria were then separated into pellet and supernantant fractions by centrifugation and western blotted for Cyt c ab (BD pharmingen). An equal amount of mitochondria not treated with tBid was used as input. **(B)** Caspase activation was monitored by immunoblotting with anti cleaved caspase3 antibody using total cell extracts from HCT116-WT BAK cells treated with or without Chk1 inhibitor for 30 min following UV. **(C)** Similarly caspase3 activation was analysed in HT1080 and HT1080-Chk1D-A (Chk1 D130A transfected in HT1080 cells) by western blotting with anti cleaved caspase 3 antibody. Actin represents equal loading.

The failure of BAK multimerization in the presence of the Chk1 inhibitor may be due to the inability of the binding of BH3 proteins. To test this idea, we next analysed whether Chk1 activity is required for release of cytochrome c from isolated mitochondria by a previously established procedure [7],[15]. Using purified recombinant BH3-activator protein tBid, we found that when tBid was incubated with mitochondria isolated from WT cells, cytochrome c was readily detected in the supernatant fractions (Figure 2A). Consistent with the multimerization results, tBid failed to release cytochrome c from mitochondria isolated from cells with prior treatment with the Chk1 inhibitor SB-218078. We then carried out caspase3 activation assays to further confirm the functional significance of cytochrome c release. Activation of caspase3 was inhibited in cells treated with the Chk1 inhibitor when compared to WT cells following UV damage (Figure 2B). In addition to these inhibitor-based assays, we also used Chk1 D130A, a kinase-dead mutant [16] in HT1080 cells to analyse caspase3 activation. We found that following UV damage WT cells were able to activate caspase3 when compared to the Chk1 D130A mutant (Figure 2C). Taken together we conclude that Chk1 activity is required for caspase3 activation and subsequent cell killing.

### The BH3-only protein PUMA is required for Chk1 mediated apoptosis

**Figure 3:**
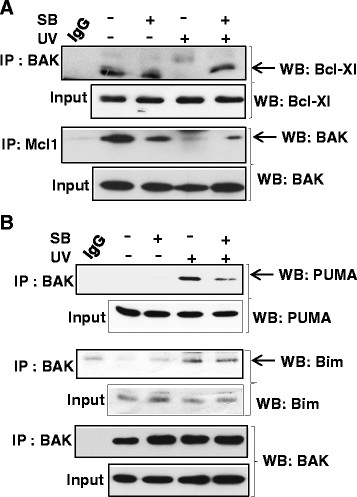
**The ability of BH3 protein and Bcl-2 family protein binding to BAK in presence of Chk1 inhibitor. (A)** Mitochondria were isolated from HCT116-WT BAK cells treated with or without Chk1 inhibitor ± prior UV. Mitochondria were then solubilised in 1% CHAPS buffer (20 mM Tris–HCl pH7.4, 135 mM NaCl, 1.5 mM MgCl2, 1 M EGTA, 10% glycerol, protease and phosphates inhibitor cocktail). Immunoprecipitations were performed with anti BAK ab (Y164, abcam) and immunoblotted for Bcl-xL (cell signalling, top panel). Similar immunoprecipitations were performed with anti MCL-1 and BAK and was detected by western blotting with rabbit anti BAK ab (Y164, abcam, bottom panel). **(B)** Similarly BAK was immunoprecipitated as described in **(A)** and immunoblotted for detection of BH3-only protein PUMA (cell signalling, top panel), Bim (Sigma, middle panel) and BAK (bottom panel). A big batch of mitochondria was isolated and aliquots were used in different immunoprecipitation reactions.

**Figure 4:**
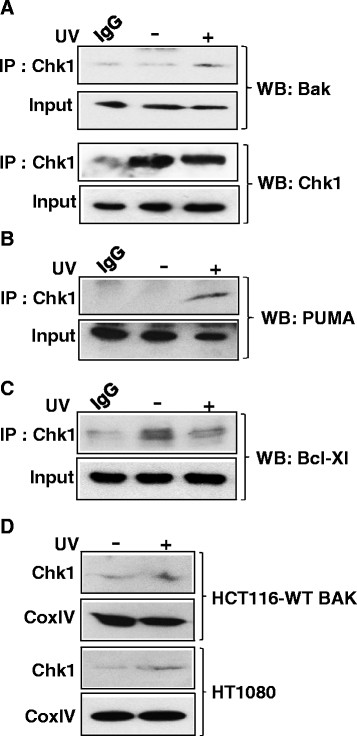
**Interaction between Chk1 and pro- and anti-apoptotic Bcl-2 family proteins. (A)** Immunoprecipitation was performed with anti-Chk1 ab (Santacruz) and immunoblotted for BAK (top panel) and Chk1 (bottom panel). Similar immunoprecipitations were performed with anti-Chk1 ab and immunoblotted for PUMA **(B)** and Bcl-xL **(C)**. In all immunoprecipitation reactions 5% of mitochondrial extracts was used as input. The non-UV mitochondrial extract was used in all IgG control immunoprecipitation reactions. **(D)** Chk1 levels were assessed in mitochondrial extracts of both HC1116-WT BAK and HT1080 cells following UV treatment or without UV. CoxIV represents equal loading.

BCL-2 family proteins, both anti- and pro-apoptotic, bind to BAK with different affinities and play an essential role in BAK activation under various stress conditions. We therefore next asked whether the Chk1 inhibitor interferes with the association between BAK and anti apoptotic proteins Bcl-xL and MCL1. Following UV damage in the presence of the Chk1 inhibitor, we found that Bcl-xL still interacts with BAK whereas, as expected, Bcl-xL dissociates from BAK upon UV damage without inhibitor. Although MCL1 is usually degraded after UV damage [17], however we were then interested to examine whether MCL1 was able to Co-IP BAK following UV irradiation in the presence of Chk1 inhibitor. We found that Mcl1 was able to pull down BAK in undamaged cells and in cells pre-treated with the Chk1 inhibitor (Figure 3A) albeit to a lesser extent. To explore further the role of Chk1, we also examined the interaction between BAK and pro-apoptotic BH3-only proteins PUMA and BIM. We found that similar levels of BIM were immunoprecipitated by BAK irrespective of whether the cells were treated with Chk1 inhibitor following UV damage. But in contrast, less PUMA was co-precipitated with BAK in the presence of the Chk1 inhibitor compared to untreated following UV damage (Figure 3B). Thus this result demonstrated that Chk1 may require PUMA for BAK activation. We next investigated the interaction between BAK and Chk1. We found that following UV damage there is an increased association between BAK and Chk1 suggesting that Chk1 is recruited to BAK during mitochondrial apoptosis upon DNA damage (Figure 4A). We also analysed the interaction between Chk1 and anti-apopototic Bcl-xL and pro-apoptotic BH3-only protein PUMA. Following UV damage there is an association between PUMA and Chk1 similar to that of BAK (Figure 4B). However, compared to UV damage there is more association of Bcl-xL with Chk1 in untreated cells (Figure 4C) further ensuring that upon UV damage, Bcl-xL dissociates from Chk1 allowing BAK and PUMA to interact, which eventually leads to BAK activation. We then assessed the presence of Chk1 in the mitochondrial preparation of both HCT116 and HT1080 cells. Following UV damage, there were increased levels of Chk1 in mitochondria from both the cell lines (Figure 4D). To our knowledge this is the first demonstration of a direct interaction between checkpoint kinase Chk1 and pro-apoptotic protein BAK.

Taken together our results now identify a novel role for Chk1 in BAK activation during mitochondrial apoptosis. Using Co-IP studies, we provide a novel direct link between Chk1 and apoptotic machinery but it remains to be established whether or not BAK is the substrate of Chk1. The BH3-only protein PUMA interferes with the binding to BAK in the presence of the Chk1 inhibitor and therefore suggests that Chk1 is responsible for the recruitment of PUMA to BAK. It was previously reported that transactivation of PUMA was restored during ATR-mediated activation of Chk1 [18]. Chk1 is regulated by PP2A and evidence for the complex feedback loop between Chk1 and PP2A has been reported [13]. PP2A activity influences many aspects of the DNA damage response. We recently showed that PP2A activity is required for BAK multimerization [7]. However, the role of Chk1 activity stimulating PP2A activity during mitochondrial apoptosis following DNA damage by UV or etoposide remains to be established. Thus, a dual role for Chk1 can be suggested. Chk1 maintains the DNA damage checkpoint integrity, however, if cells have sustained severe DNA damage and are no longer repairable, Chk1 may then play a role in apoptosis by interacting with BAK. Recently it has been reported that Chk1 is activated by caspase-dependent cleavage during apoptosis [19]. In response to DNA damage Chk1 becomes phosphorylated on several serine residues (S280, S296, S317 and S345) and plays important functions in cellular processes. Thus, it would be important to investigate the role of this phosphorylation in BAK activation. Moreover, in this study we used mostly HCT116 cells which is lacking mismatch repair and Chk1 is also involved in mismatch repair (mmr). However our finding is not due to the abnormalities in mmr rather due to the inhibition of Chk1 activity as we found that caspase3 activation is also inhibited in HT1080 cells. Finally, Chk1 is may be responsible for a providing a safe guarding mechanism for the cell, so that it is poised to undergo apoptosis if DNA damage is detected and cannot be repaired.

## Competing interests

The authors state that they have no competing interests.

## Authors’ contributions

AA designed the experiments; AA performed all experimental procedures and generated images; AA analysed data and prepared the manuscript. Both authors read and approved the final manuscript.
